# Validity and Reliability of Gait Speed and Knee Flexion Estimated by a Novel Vision-Based Smartphone Application

**DOI:** 10.3390/s24237625

**Published:** 2024-11-28

**Authors:** Kam Lun Leung, Zongpan Li, Chen Huang, Xiuping Huang, Siu Ngor Fu

**Affiliations:** Department of Rehabilitation Sciences, The Hong Kong Polytechnic University, Hong Kong, China; aaron.leung@polyu.edu.hk (K.L.L.); po.zp.li@connect.polyu.hk (Z.L.); cece.huang@connect.polyu.hk (C.H.); phoebe-xp.huang@connect.polyu.hk (X.H.)

**Keywords:** smartphone application, walking speed, knee flexion, knee osteoarthritis, fall

## Abstract

Patients with knee osteoarthritis walk with reduced speed and knee flexion excursion in the early stance phase. A slow walking speed is also associated with falls in older adults. A novel vision-based smartphone application could potentially facilitate the early detection of knee osteoarthritis and fall prevention. This study aimed to test the validity and reliability of the app-captured gait speed and peak knee flexion during the initial stance phase of gait. Twenty adults (aged 23–68 years) walked at self-selected comfortable walking speeds while the gait speed and knee flexion were simultaneously measured using retroreflective sensors and Xsens motion trackers and the app in two separate sessions for validity and reliability tests. Pearson’s *r* correlation and Bland–Altman plots were used to examine the correlations and agreements between the sensor- and app-measured outcomes. One-sample *t*-tests were performed to examine whether systematic bias existed. The intraclass correlation coefficient (ICC) was calculated to assess the test–retest reliability of the app. Very high correlations were found between the sensor and app measurements for gait speed (*r* = 0.98, *p* < 0.001) and knee flexion (*r* = 0.91–0.92, all *p* < 0.001). No significant bias was detected for the final app version. The app also showed a good to excellent test–retest reliability for measuring the gait speed and peak knee flexion (ICC = 0.86–0.94). This vision-based smartphone application is valid and reliable for capturing the walking speed and knee flexion during the initial stance of gait, potentially aiding in the early detection of knee osteoarthritis and fall prevention in community living locations.

## 1. Introduction

With the increasing population and human lifespan, aging has led to significant challenges globally [1]. Knee osteoarthritis is a prevalent chronic condition, affecting 37% of individuals aged 60 and older [2]. Falls are also a major concern in the aging population, with 29% of community-dwelling adults aged 65 and older experiencing at least one fall yearly [3]. Knee osteoarthritis can lead to pain [4], muscle weakness [5], and limitations in joint range of motion [6], consequently increasing the risk of falls [7], injury, anxiety, depression, and loss of independence [8]. Falls are a leading cause of injury and morbidity among older adults [9]. The fear of falling can lead to reduced physical activity and social isolation, further aggravating the risk of falls and declining health [10]. These factors place great demands on public health resources.

Patients with knee osteoarthritis walk with reduced speed [11], greater knee flexion at heel strike [11,12,13,14], and less knee flexion excursion during the initial stance [13,14]. Lower peak knee flexion during the loading response has been linked to a reduced knee flexion moment and higher sagittal knee dynamic joint stiffness, which are associated with an elevated risk of developing chronic knee pain and clinical OA in older adults [15,16]. Therefore, monitoring the peak knee flexion during the loading response phase of gait could serve as a valuable screening tool for identifying individuals at risk of knee pain and OA in the aging population. A self-selected walking speed below 1.0 m/s was associated with a history of multiple falls in older adults living in residential care [17]. Slowing the gait speed over 12 months was associated with increased risk of one or more falls [10]. A gait assessment of the walking speed and dynamic knee flexion angle can thus be used for the early detection or identification of individuals prone to knee osteoarthritis and falls, providing appropriate management to maintain the functional ability and enable healthy aging.

Three-dimensional gait analysis systems are regarded as the gold standard for motion analysis but their expensive cost limits the accessibility to specialized laboratory setting [18]. Automated markerless 2D video-based gait analysis systems were developed. The moving human body and its contour were detected from image sequences to extract gait figures with joint angles and body points for the analysis of motion [19,20]. Although the setup time is reduced and the equipment is easy to use, it may not achieve the same level of accuracy and precision as marker-based systems, especially for fine or subtle movements [21]. Wearable motion tracking systems are recognized as reliable methods for tracking human movement, but their usage is hindered by the relatively high cost of the sensor itself and the associated processing unit [22]. Moreover, sensor accuracy is affected by drift and comfort issues [23]. Smartphone gait applications incorporating wearable sensors have been developed to measure gait parameters. The subjects wear external sensors, such as an accelerometer, gyroscope, inclinometer, and goniometer or use the smartphone’s on-board accelerometer to assess the walking distance, step timing, gait symmetry, joint angles, vertical center of mass displacement, and other metrics [24,25,26,27,28].

Healthy aging involves not only the absence of disease, but also the process of developing and maintaining the functional ability that enables well-being [29]. “Aging-in-place” has been regarded as the cornerstone of aged care policy and has the advantages of allowing older people to live and age in an environment with a sense of attachment and a feeling of security [30]. The use of technology in community living locations is a major step in promoting “aging-in-place” [31,32]. Since the smartphone has become an integral part of daily life, it would be practical and convenient for seniors and healthcare workers in the community to use it to assess and monitor health status, thereby promoting healthy aging. The privacy concerns associated with using the smartphone application for data collection remain a barrier to its widespread use. Mobile apps are vulnerable to data breaches, which can expose sensitive user information to unauthorized parties. Privacy concerns are a significant issue when it comes to mobile app-collected data, including data from gait analysis apps. Ensuring the secure storage and transmission of data is crucial to protect user privacy [33].

A novel vision-based smartphone gait application has been developed to apply computer vision pose estimation techniques [34] to locate points of interest, such as the knee joint, on the human body from a single RGB camera-captured two-dimensional video to estimate the walking speed and sagittal plane knee motions. The app, supported by a backend analyzer program, is intended to offer functional screening and recommendations and facilitate continuous assessment for the early detection and monitoring of knee osteoarthritis and falls in older adults living in a community. This project aimed to test the validity and reliability of the mobile app by comparing the smartphone-estimated walking speed and knee flexion data to those measured by retroreflective sensors and wearable motion trackers.

## 2. Materials and Methods

### 2.1. Participants

Twenty subjects were recruited for the validity and reliability tests. The inclusion criteria included adults who could walk independently. Participants were excluded if they were unable to walk independently; had undergone knee surgery within the previous year; had joint pain or an injury of the lower limb, such as the ankle, knee, or hip, within the previous three months; or were not able to complete the test. The study was approved by the human subject ethics subcommittee of the administering institution (Reference number: HSEARS20220527006). All eligible participants provided written informed consent before data collection. Demographic data including age sex, body height, and body mass were requested and recorded.

### 2.2. Equipment

**Figure 1:**
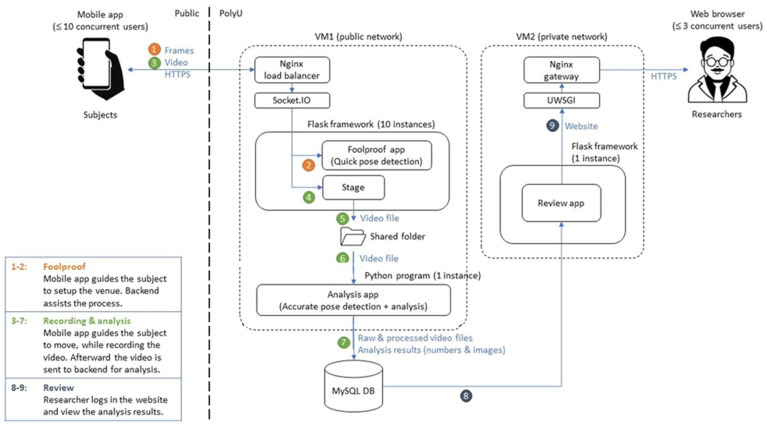
System diagram.

An android-run smartphone with a single RGB camera was installed with the app to capture video for analysis (Figure 1). An Ngnix load balancer (Ngnix, San Francisco, CA, USA) in backend public network server 1 (VM1) can handle multiple mobile phone connections simultaneously. The mobile app provides on-screen guidelines and verbal instructions for the tester to capture motion and send the file frame by frame in real time to the Flask framework to start quick pose detection. To ensure data will be captured correctly, VM1 will implement foolproof capabilities and will be included in the mobile app to warn if the subject is out of view or too far away, or not walking perpendicularly to the optical axis the smartphone.

Afterward, the video will be sent to the Python program (version 3.10.0) of VM1 for accurate post-detection and analysis. The raw and processed video files along with analysis results in numerical and image formats will be stored in a MySQL database. The researcher can log in to the website of Backend server 2 (VM2) to review the analysis results. The results can also be disseminated through an Nginx gateway.

The use of retroreflective markers in motion capture systems for measuring human speed and other gait parameters have been validated [35]. The Xsens IMU-based system showed high accuracy for flexion/extension joint angles but lower correlation for other joint axes [36]. It is highly accurate for measuring knee flexion angles [37,38]. Since the smartphone app only extracted knee flexion angle for assessment, Xsens and retroreflective sensors (E3JK-DS30M1) were used to test the validity and reliability of the smartphone-estimated knee flexion angles and walking speed.

### 2.3. Equipment Setup

**Figure 2:**
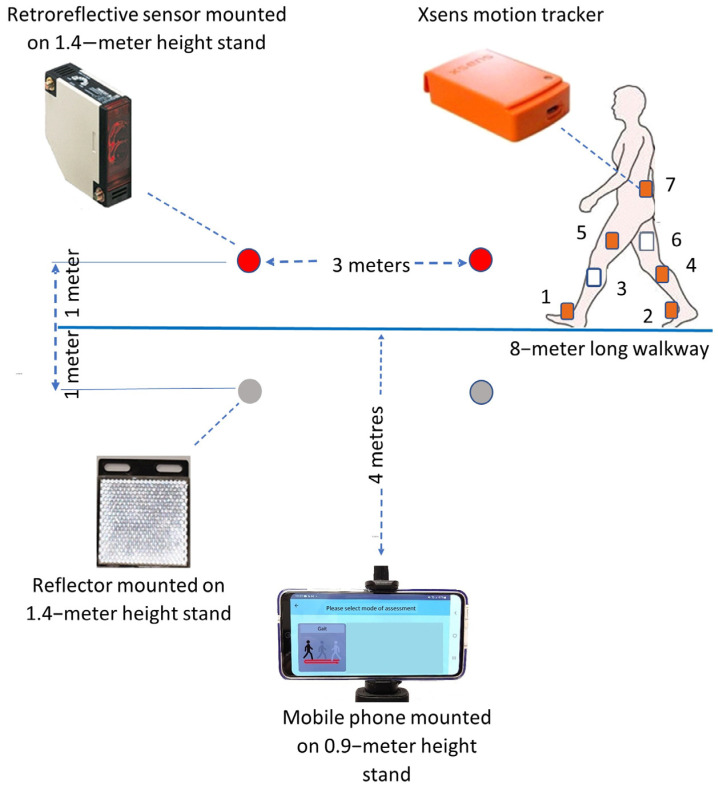
Layout for the validity and reliability tests of the smartphone application. Seven X-sens motion tracker (numbers 1–7) are placed on the lower limbs of the subject: (1) dorsal surface of left foot, (2) dorsal surface of right foot, (3) medial surface of left tibia, (4) medial surface of right tibia, (5) left lateral thigh above the knee joint, (6) right lateral thigh above the knee joint, (7) pelvis.

The smartphone was mounted on a 0.9–meter−high stand at a perpendicular distance of 4 m away from the midpoint of an 8−meter walkway (Figure 2). At each end of the central 3 m of the walkway, a retroreflective sensor and a reflector were separately mounted on 1.4−meter−high stands and placed 2 m apart. Seven Xsens MVN Awinda system wearable motion trackers were placed on the dorsal surface of the left (1) and right (2) midfoot, the medial surface of the left (3) and right (4) tibia, the left (5) and right (6) lateral thighs above the knee joint, and the pelvis (7) of each subject to validate knee joint kinematics.

### 2.4. Experimental Procedures

The smartphone-estimated results were compared with data measured by a wearable motion tracker system and a retroreflective sensor system for validation. The equipment was set up as shown in Figure 2. Each subject, with wearable motion trackers attached to body segments, was firstly guided to perform walking activities to complete the motion tracker calibration process. For video capture, each subject was firstly required to follow the app-provided verbal instruction to face the smartphone camera and stand erect. The image was sent to VM1 for verification. The subject was then required to walk at a self-selected comfortable walking speed from one end to the other end of the walkway for one cycle. Two experiments were conducted on the same day to evaluate test–retest reliability. A 10 min rest was arranged between 2 tests.

### 2.5. Statistical Analysis

Pearson’s *r* correlation tests were used to examine the correlations between the sensor- and the app-measured walking speed and knee flexion angle during the loading response phase of gait. The correlation coefficients (*r*) were interpreted as follows: 0.90–1.0 (very high), 0.70–0.90 (high), 0.50–0.70 (moderate), 0.50–0.70 (low), or 0.00–0.30 (negligible) correlation [39]. Bland–Altman plots were generated to verify the agreement between the app and sensors for the measurements of walking speed and knee flexion angle. For each measurement, the mean difference, percentage of the mean difference, and 95% limits of agreement were reported. We also performed a one-sample *t*-test for the mean differences to examine whether systematic bias existed.

The intraclass correlation coefficient model (2, 1) (ICC _(2, 1)_) with a 95% confidence interval (95% CI) was calculated to assess the test–retest reliability of the app-measured walking speed and knee flexion angle. An ICC _(2, 1)_ of >0.90 was considered as excellent, 0.75–0.90 as good, 0.50–0.75 as moderate, and < 0.50 as poor test–retest reliability [40]. Based on the reliability coefficients, the standard error of measurement (SEM) was calculated as (SD × √1-ICC) for each measurement. The minimal detectable difference (MDD_95_) was computed as 1.96 × SEM × √2 (for a 95% CI) [41]. Data analyses were performed in SPSS (Version 23.0, IBM Corp., Armonk, NY, USA). Statistical significance was set at *p* < 0.05.

## 3. Results

### 3.1. Participants

A total of 20 healthy adults were included in this study (55% females, age: 35.5 ± 12.8 years). The demographics of these participants are presented in Table 1.

### 3.2. Validity of Walking Speed

**Figure 3:**
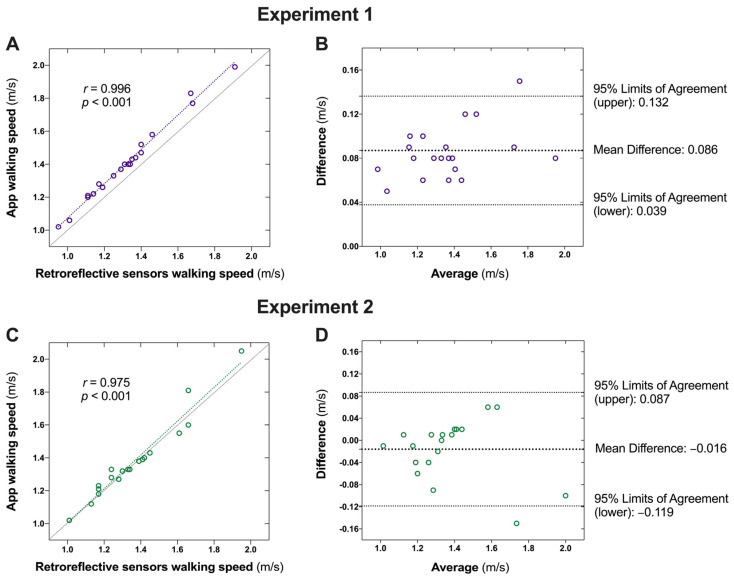
Agreement between the retroreflective sensor- and the app-captured walking speed. (**A**): Correlation between retroreflective sensor- and the app-captured walking speed in Experiment 1; **(B**) Agreement between the retroreflective sensor- and the app-captured walking speed in Experiment 1; (**C**): Correlation between retroreflective sensor- and the app-captured walking speed in Experiment 2; (**D**) Agreement between the retroreflective sensor- and the app-captured walking speed in Experiment 2.

The results of the two experiments for the validity tests of walking speed are presented in Table 2. In experiment one, a very high correlation was found between the retroreflective sensor-measured speed and the app-measured walking speed (*r* = 0.996, *p* < 0.001; Figure 3A). However, a systematic bias was observed, with the app-measured walking speed being consistently higher than the sensor-measured walking speed (bias = 0.086 m/s, *p* < 0.001; Figure 3B). We then modified the algorithm by subtracting the systematic bias and conducted the second validity test following the same experimental protocol with the same participants. The results revealed a very high correlation between the sensor- and app-measured walking speeds (*r* = 0.975, *p* < 0.001; Figure 3C). The Bland–Altman plots with one-sample t-tests indicated no significant systematic bias for the app with the adjusted algorithm (mean difference: −0.016 ± 0.052 m/s, 95% limits of agreement: −0.119–0.087 m/s, *p* = 0.281; Figure 3D).

### 3.3. Validity of the Knee Flexion Angle

**Figure 4:**
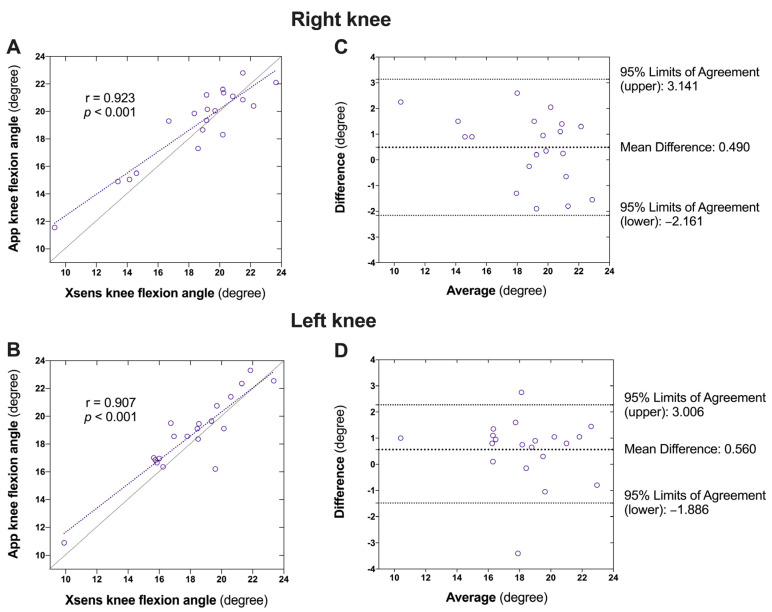
Agreement between the Xsens motion tracker- and the app-captured peak knee flexion during initial stance phase of gait. (**A**): Correlation between the Xsens motion tracker- and the app-captured peak knee flexion of the right knee; (**B**) Agreement between the Xsens motion tracker- and the app-captured peak knee flexion of the right knee; (**C**): Correlation between the Xsens motion tracker- and the app-captured peak knee flexion of the left knee; (**D**) Agreement between the Xsens motion tracker- and the app-captured peak knee flexion of the left knee.

Very high correlations were observed between the sensor-measured and app-measured knee flexion angles for both the right (*r* = 0.923, *p* < 0.001; Figure 4A) and left (*r* = 0.907, *p* < 0.001; Figure 4B) knees. The Bland–Altman plots with one-sample *t*-tests also revealed no significant bias of the app compared with the sensor-captured knee flexion angle on either the right (mean difference: 0.490 ± 1.353°, 95% limits of agreement: −2.161 to 3.141°, *p* = 0.122; Figure 4C) or left (mean difference: 0.560 ± 1.248°, 95% limits of agreement: −1.886° to 3.006°, *p* = 0.059; Figure 4D) knee (Table 3).

### 3.4. Reliability of the App for Walking Speed and Knee Flexion Angle

The test–retest reliabilities of the app-captured walking speed and knee flexion angle are summarized in Table 4. The app showed an excellent test–retest reliability for measuring the walking speed (ICC _(2, 1)_ [95% CI]: 0.94 [0.84, 0.97], SEM: 0.05, MDD: 0.20) and a good test–retest reliability for capturing the right (ICC _(2, 1)_ [95% CI]: 0.86 [0.63, 0.95], SEM: 0.77–0.91, MDD: 3.02–3.57) and left (ICC _(2, 1)_ [95% CI]: 0.94 [0.84, 0.97], SEM: 0.71–0.80, MDD: 2.78–3.14) knee flexion angles.

## 4. Discussion

This study examined the validity and test–retest reliability of an inexpensive and easy-to-operate method to assess the spatiotemporal and knee kinematic parameters of gait. The accuracy of the retroreflective sensors depends on various factors, including the placement of the markers, the calibration of the system, and the environmental conditions [42].

A significant positive correlation was discovered between the walking speed measurements obtained with the app and those obtained with the retroreflective sensors. However, the initial algorithm within the app consistently registered a slightly higher speed than the retroreflective sensors registered (by 0.086 m/s). The algorithm was fine-tuned to correct this systematic bias. After recalibration, the experiments were conducted again with the same group of participants and protocol. The systematic bias was eliminated, and a strong correlation persisted between the app- and retroreflective sensor-measured walking speeds. A study on a human pose estimation model for measuring single-leg squat kinematics [43] reported strong relationships (*r* = 0.94) between the 2D pose estimation and 3D motion analysis for knee flexion and moderate relationships (*r* = 0.68) for ankle dorsiflexion. The Bland–Altman analysis showed a 1.2° mean difference for knee flexion and −14.5° mean difference for ankle dorsiflexion. The knee flexion angle measured at the initial loading response by the app also demonstrated a strong correlation with that measured with the Xsens motion sensors.

Some smartphone apps developed for gait measurement require the subjects to mount the phone on their body [44,45] for data collection. Various smartphone apps have been developed to assess joint motion [46,47]. A video goniometer app was introduced for measuring joint angles during functional activities. However, this app requires the manual labeling of anatomical points from the video by external coders [48]. In contrast, our app incorporates a built-in algorithm capable of automatically detecting anatomical points and capturing dynamic knee angles from video footage. This newly developed smartphone app empowers individuals to record sample videos at home while receiving real-time feedback on their walking performance from the app. Moreover, it accurately identifies the peak knee flexion angle during the loading response phase of gait, a critical feature for individuals with knee osteoarthritis who typically exhibit reduced knee flexion angles during this phase of gait [49,50].

The significance of this app lies in its ability to offer a sensor-free and cost-effective method for individuals to record simple videos in the community, such as at home, while receiving real-time results on the walking speed and peak knee flexion angle during the loading response phase of gait. These metrics are associated with frailty, knee osteoarthritis, an increased risk of falls, and other adverse health such as cognitive decline, cardiovascular disease, cancer, and functional dependence among the aging population [51,52,53,54,55]. The results demonstrated that the app is a valid tool to capture the walking speed and dynamic knee flexion angle with a good to excellent test–retest reliability. The mobile app system can be used and the results can be read by any person without a health background and community healthcare workers. In addition to knee osteoarthritis and falls, healthcare professionals can also utilize the app system to monitor the health status that can be reflected by the walking speed and lower limb kinematics. The early detection of knee osteoarthritis and falls offers a significant positive impact on individuals and healthcare systems, including an improved quality of life, the prevention of disease progression and injuries, cost savings, and enhanced independence.

One limitation arose from instances where participants wearing dark-colored pants experienced occasional confusion of pose estimation, affecting the calculation of the right and left knee angles. Changes in lighting, background, and other environmental factors can affect the performance of markerless systems, as they rely on visual data to track movements [56]. Appropriate arrangements should be made to mitigate this issue. Refining the built-in algorithm would bolster the screening capabilities. In addition to the technical capability addressed, the analysis of privacy concerns in mobile app gait analysis requires a multifaceted approach. This includes transparent data collection, storage, and sharing; strong security measures; strict data protection regulations; and user control [57]. To mitigate the risk of a data breach, the application stored data on a university central authentication server operated by the Information Technology Services Department. The department provided adequate protection against threats like malware, phishing, and hacks through regularly updating software and systems; deploying firewalls and antivirus software; and conducting regular security audits. Only authorized persons with the administrator password could access the master data set for evaluation and research. Individual subjects could only view their own data stored on the website using their individual passwords.

For future direction, the developed protocol can be utilized as a triage method to classify the tested subjects into normal (within one standard deviation), marginal (within one to two standard deviations), and unhealthy (beyond two standard deviations) groups. An appropriate exercise program will be prescribed for these three groups of subjects. Those subjects with scores beyond two standard deviations will be advised to seek medical advice. The subjects who participated in an exercise program will be re-assessed with the app system for outcome evaluation. We anticipate that this innovative app will contribute significantly to promoting “aging-in-place” practices within general communities, thereby enhancing the overall well-being and quality of life.

## 5. Conclusions

In conclusion, this novel app has been demonstrated to be a valid and reliable tool for capturing the walking speed and dynamic knee flexion angle during gait, with good to excellent test–retest reliability. It could be a potential tool for the early detection of knee osteoarthritis and fall prevention in general communities, thereby promoting better practices of “aging-in-place” and “healthy aging”.

## Figures and Tables

**Table 1:** Demographics.

	*n* = 20
Age (years)	35.5 ± 12.8
Sex (*n*, % of females)	11, 55%
Body height (m)	1.7 ± 0.1
Body mass (kg)	60.5 ± 14.5
BMI (kg/m^2^)	21.9 ± 3.3

BMI: body mass index.

**Table 2:** Validity of the app-captured walking speed.

	Measurements		Correlation		Agreement			
	Retroreflective Sensors (m/s)	APP (m/s)	*r*	*p*	Difference (%)	Difference (m/s)	95% Limits of Agreement (m/s)	*p* ^ †^
Experiment 1	1.32 ± 0.23	1.41 ± 0.24	0.996	<0.001	6.5	0.086 ± 0.024	(0.039, 0.132)	<0.001
Experiment 2	1.38 ± 0.23	1.36 ± 0.22	0.975	<0.001	−0.7	−0.016 ± 0.052	(−0.119, 0.087)	0.281

Values are mean ± SD unless otherwise indicated; **^†^** *p* values from one-sample *t*-tests.

**Table 3:** Validity of the app-captured peak knee flexion during initial stance.

	Measurements		Correlation		Agreement			
	Xsens (Degree)	APP (Degree)	*r*	*p*	Difference (%)	Difference (Degree)	95% Limits of Agreement (Degree)	*p* ^ †^
Right knee	18.8 ± 2.8	19.1 ± 2.7	0.923	<0.001	2.6	0.490 ± 1.353	(−2.161, 3.141)	0.122
Left knee	18.9 ± 3.9	19.8 ± 3.8	0.907	<0.001	3.2	0.560 ± 1.248	(−1.886, 3.006)	0.059

Values are mean ± SD unless otherwise indicated; **^†^** *p* values from one-sample *t*-tests.

**Table 4:** Test–retest reliabilities of the app for walking speed and peak knee flexion during initial stance of gait.

			Mean ± SD (Degree)	SEM (Degree)	MDD_95_ (Degree)	ICC _(2, 1)_ (95% CI)
Walking speed		Test 1	1.35 ± 0.22	0.05	0.20	0.94
		Test 2	1.38 ± 0.23	0.05	0.20	(0.84, 0.97)
Knee flexion	Right knee	Test 1	18.1 ± 3.5	0.91	3.57	0.86
		Test 2	19.3 ± 3.0	0.77	3.02	(0.63, 0.95)
	Left knee	Test 1	17.7 ± 3.1	0.80	3.14	0.88
		Test 2	18.9 ± 2.7	0.71	2.78	(0.69, 0.95)

ICC: intraclass correlation coefficient; CI: confidence interval; SD: standard deviation; SEM: standard error of measurement; MDD_95_: minimum detectable difference (for a 95% CI).

## Data Availability

The data presented in this study are available on request from the corresponding author due to privacy considerations.
